# A potential anti-tumor herbal medicine, Corilagin, inhibits ovarian cancer cell growth through blocking the TGF-β signaling pathways

**DOI:** 10.1186/1472-6882-13-33

**Published:** 2013-02-15

**Authors:** Luoqi Jia, Hongyan Jin, Jiayi Zhou, Lianghua Chen, Yiling Lu, Yanlin Ming, Yinhua Yu

**Affiliations:** 1 Department of Gynecology, Obstetrics and Gynecology Hospital of Fudan University, Shanghai Key Laboratory of Female Reproductive Endocrine Related Diseases, Shanghai 200011, People’s Republic of China; 2 The Research and Development Center for Medicine Plants and Plant Drugs, Xiamen Overseas Chinese Subtropical Plant Introduction Garden, Xiamen, 361002, People’s Republic of China; 3 The University of Texas, M.D. Anderson Cancer Center, Houston, TX, 77030, USA

**Keywords:** Corilagin, Herbal medicine, TGF-β, Ovarian cancer, Epithelial-mesenchymal transition, Cell cycle G2/M arrest, Apoptosis

## Abstract

**Background:**

*Phyllanthus niruri* L. is a well-known hepatoprotective and antiviral medicinal herb. Recently, we identified Corilagin as a major active component with anti-tumor activity in this herbal medicine. Corilagin is a member of the tannin family that has been discovered in many medicinal plants and has been used as an anti-inflammatory agent. However, there have been few reports of the anti-tumor effects of Corilagin, and its anti-tumor mechanism has not been investigated clearly. The aim of the present study is to investigate the anticancer properties of Corilagin in ovarian cancer cells.

**Methods:**

The ovarian cancer cell lines SKOv3ip, Hey and HO-8910PM were treated with Corilagin and analyzed by Sulforhodamine B (SRB) cell proliferation assay, flow cytometry, and reverse phase protein array (RPPA). Corilagin was delivered intraperitoneally to mice bearing SKOv3ip xenografts.

**Results:**

Corilagin inhibited the growth of the ovarian cancer cell lines SKOv3ip and Hey, with IC50 values of less than 30 μM, while displaying low toxicity against normal ovarian surface epithelium cells, with IC50 values of approximately 160 μM. Corilagin induced cell cycle arrest at the G2/M stage and enhanced apoptosis in ovarian cancer cells. Immunoblotting assays demonstrated that Cyclin B1, Myt1, Phospho-cdc2 and Phospho-Weel were down-regulated after Corilagin treatment. Xenograft tumor growth was significantly lower in the Corilagin-treated group compared with the untreated control group (*P* <0.05). More interestingly, Corilagin inhibited TGF-β secretion into the culture supernatant of all tested ovarian cancer cell lines and blocked the TGF-β-induced stabilization of Snail. In contrast, a reduction of TGF-β secretion was not observed in cancer cells treated with the cytotoxic drug Paclitaxel, suggesting that Corilagin specifically targets TGF-β secretion. Corilagin blocked the activation of both the canonical Smad and non-canonical ERK/AKT pathways.

**Conclusions:**

Corilagin extracted from *Phyllanthus niruri L.* acts as a natural, effective therapeutic agent against the growth of ovarian cancer cells via targeted action against the TGF-β/AKT/ERK/Smad signaling pathways.

## Background

Ovarian cancer is the most common form of gynecologic neoplasm and the fifth most common cause of cancer mortality in women. Although there have been improvements in surgical techniques and treatment options, the five-year survival for stages IIB to IV ovarian cancer is less than 40% [1]. The current chemotherapeutic in common clinical use is platinum combined with Paclitaxel, which has enhanced drug toxicity. Therefore, researchers are searching for new anti-ovarian cancer drugs that are eutherapeutic and inflict fewer side effects. Work in herbal medicine is especially highlighted.

Since 2005, we have screened hundreds of herbs, among which *Phyllanthus niruri* L. has the greatest anti-cancer potential. *Phyllanthus niruri* L. (*P. niruri* L.) belongs to the Euphorbiaceae family and originated in India. It usually occurs as a winter weed throughout the tropic and subtropic parts of the globe, including China, South Asia, and America. Our garden has introduced and domesticated this plant since the 1960s. In this study, whole *P. niruri* plants were collected from Gulangyu Islet, Fujian province, China, in October 2006 and identified by Professor Yong-Tian Zhang, Fujian Institute of Subtropical Botany, China. A voucher specimen (20061026) was deposited at Xiamen Overseas Subtropical Plant Introduction Garden, China. *P. niruri* L. is a popular folk medicine for treating nephritic, urocystic, gastrointestinal, and hepatic infections. It has traditionally been used in antiviral, antioxidant, anti-inflammatory, and antidiabetic treatments as well as for radiation protection. Our recent work identified that Corilagin is a major active compound from *P. niruri* L. extracts; it is effective in retarding the growth of hepatocarcinoma cells [unpublished data, Ming *et al*.].

There has been little research on the effect of Corilagin on cancer; much of the current research on Corilagin focuses on its use as an antiviral, hypolipemic, hypotensive and anticoagulation agent [2,3]. A study from Hau DK*et al.* showed that Corilagin is considerably effective at retarding the *in vivo* growth of xenografted Hep3B hepatocellular carcinoma cells [4]; however, there are few reports on the pharmacology and molecular mechanism of Corilagin. When screening plant extracts for TNF-α inhibitors, Okabe *et al.*[5] and Fujiki *et al.*[6] found that Corilagin could significantly inhibit the secretion of TNF-α.

In this study, we investigated the effect of Corilagin on ovarian cancer cells both *in vitro* and *in vivo*. We further explored the intracellular mechanisms involving Corilagin in multiple signaling pathways and in inflammatory factor secretion.

## Methods

### Cell culture and reagents

The human ovarian cancer cell lines SKOv3ip and Hey were obtained from the M. D. Anderson Cancer Center (Houston, TX, U.S.A.). HO8910PM, a highly metastatic ovarian cancer cell line [7], was obtained from the Chinese Academy of Sciences (Shanghai, China). These cell lines were cultured in DMEM or RPMI 1640 medium supplemented with 10% fetal bovine serum. To study the correlation of Snail and TGF-β, we transfected the Snail expression vector into HO8910PM cells, thereby producing a stable Snail-expressing cell line, which was cultured in RPMI 1640 medium supplemented with 10% fetal bovine serum and 400 μg/ml of G418. Nonmalignant ovarian surface epithelial (OSE) cells were obtained by lightly scraping the ovarian epithelial surface, followed by culture in medium 199:105 supplemented with 15% fetal bovine serum and 10 ng/ml EGF (Sigma, St. Louis, MO), as previously described [8]. All samples were obtained with the patient’s informed consent using protocols and procedures approved by the Institutional Review Board at the Obstetrics and Gynecology Hospital of Fudan University. The antibodies against pAKT, AKT, pERK, ERK and Snail and the Cell Cycle Regulation Antibody Sampler Kit II were purchased from Cell Signaling Technology (Danvers, MA), and an anti-GAPDH antibody was purchased from Kang Chen Bio Co. (Shanghai, China). TGF-β1 was purchased from Sigma.

### Extraction and purification of corilagin

**Figure 1:**
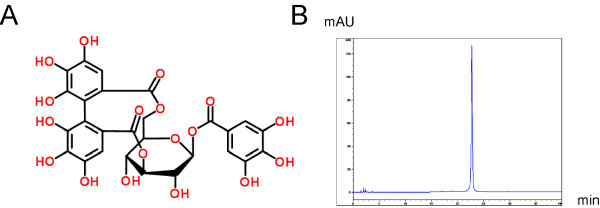
**(A) A diagram of the structure of Corilagin. (B) HPLC confirmation of the purity of Corilagin.**

Corilagin was extracted and purified by the Xiamen Overseas Chinese Subtropical Plant Introduction Garden. Dried, whole *Phyllanthus niruri* L. herb was extracted three times with ethanol, then with n-hexane, trichloromethane ethyl acetate, and n-butanol successively. The n-butanol fraction was subjected to Medium Pressure Liquid Chromatography (MPLC) using 5% (*v/v*) acetone for washes and 15% (*v/v*) acetone for elution. The fraction obtained from the 15% acetone elution was subjected to a polyamide column using 15% (*v/v*) ethanol to wash, then 25% (*v/v*) ethanol to elute. The fraction obtained from the 25% ethanol elution was subjected to a Sephadex LH-20 column to yield Corilagin. The purity of Corilagin reached 98.7%, which was confirmed by High Performance Liquid Chromatography (HPLC) (Figure 1).

### Cell proliferation assay

Sulforhodamine B (SRB) was used to detect the effect of drugs on the proliferation of ovarian cancer cell lines and OSE cells. Cancer cells (1.5-2.0×10^3^ cells per well in 100 μl medium) and OSE cells (3.0×10^3^ cells per well in 100 μl medium) were seeded in 96-well plates and incubated with Corilagin (0, 8, 16, 24, 32, or 40 μM for the cancer cells and 0, 60, 120, 240, or 480 μM for the OSE cells) starting the following day and continuing for 3 days. After 72 hours, 50 μl of 30% trichloroacetic acid was added and incubated for 60 min at 4°C. After washing and drying the plate, 100 μl of 0.4% SRB was added for 30 min. The plates were rinsed with 0.1% acetic acid and air-dried, after which 100 μl of Tris base (10 mM/L) was added, and the plates were shaken for 5 min. The SRB value was measured at a wavelength of 490 nm. The experiment was performed in quintuplicate and repeated three times.

### Cell cycle analysis

SKOv3ip and Hey cells were seeded in 60-mm plates (1–2×10^5^/plate) and incubated with Corilagin (20–40 μM) or DMSO as a control the next day. Control and treated cells were trypsinized at 24 or 48 hours after treatment, collected in PBS and fixed on ice, followed by washing with 70% cold ethanol. After treatment with 10 μg/ml RNase, cells were stained with 50 μg/ml propidium iodide (PI, Sigma) for 15 min at room temperature in preparation for cell cycle analysis. Stained cells were analyzed by flow cytometry (FACS Calibur) (BD, Franklin Lakes, NJ). The cell cycle information was analyzed using ModFit3.0 software.

### Apoptosis analysis

Hey cells were seeded in a 60-mm dish (1–2×10^5^/dish) and incubated with Corilagin (20–40 μM) or DMSO as a control. Control and treated cells were trypsinized at 24 and 48 hours, collected in PBS and stained with Annexin V and PI according to the manufacturer’s instructions for the Vybrant ^®^ Apoptosis Assay Kit (Invitrogen). The stained cells were analyzed by flow cytometry.

### Reverse phase protein array (RPPA) analysis

Untreated and Corilagin-treated HO8910PM cells were used for RPPA analysis at The University of Texas, M.D. Anderson Cancer Center RPPA Core Facility. We followed the methods described at the following web address: http://www.mdanderson.org/education-and-research/resources-for-professionals/scientific-resources/core-facilities-and-services/functional-proteomics-rppa-core/index.html.

### Western blot analysis

SKOv3ip cells and Hey cells were seeded in 60-mm plates (1–2×10^5^/plate) and incubated with Corilagin (20–40 μM) or DMSO, as a control, for 24, 48 or 72 hours. Cell lysates were harvested with lysis buffer (1% Triton X-100, 50 mM Hepes, pH 7.4, 150 mM NaCl, 1.5 mM MgCl_2_, 1 mM EGTA, 100 mM NaF, 10 mM NaPPi, and 10% glycerol, to which 1 mM PMSF, 1 mM Na_3_VO_4_, and 1X protease inhibitor were added before use). HO8910PM-snail cells were seeded in a 60-mm plate and treated with TGF-β1 alone or in combination with Corilagin; DMSO was used as the control.

Proteins from total cell lysates were separated using a 10-15% SDS-PAGE gel and transferred to PVDF membranes (Millipore, Billerica, MA). The membranes were blocked, washed and incubated with specific primary antibodies. The primary antibody incubation was followed by incubation with HRP-conjugated secondary antibodies. The bands were detected with an enhanced chemiluminescence assay (PerkinElmer, Waltham, MA).

### ELISA

Various ovarian cancer cell lines were seeded in 60-mm plates (1-3×10^5^/plate) and incubated with Corilagin (20, 40, or 80 μM) or DMSO. Culture supernatants were harvested after 1, 2, and 3 days to measure the concentration of TGF-β1. Hey cells (2.0×10^3^ cells per well in 100 μl of medium) were seeded in 96-well plates and incubated with Corilagin (40 μM), Paclitaxel (40 nM), or DMSO the next day. Culture supernatants were harvested at 48 h to measure the concentration of TGF-β1. SRB was used to detect the effects of Corilagin and Paclitaxel on the proliferation of ovarian cancer cells. The concentration of TGF-β1 was measured by ELISA according to the manufacturer’s instructions (Shanghai ExCell Biology, Inc., Shanghai, China).

### Growth of xenografts in nu/nu mice

All animal experiments were carried out in accordance with an animal protocol approved by the Institutional Animal Care and Use Committee of the Shanghai Tumor Institute. The effect of Corilagin on the *in vivo* growth of ovarian cancer xenograft tumors was evaluated using xenografts of the human ovarian cancer cell line SKOv3ip in Balb/c nu/nu mice. The SKOv3ip cells (1.5 × 10^6^) were injected subcutaneously. Tumors were measured twice a week, and tumor volumes were calculated using the formula TV = (L × W^2^)/2, where L represents the longer diameter and W represents the shorter diameter. When palpable tumors had grown to a diameter of 0.3-0.5 cm, the mice were divided into four groups of six to eight, and each group received an intraperitoneal injection of either DMSO (as a control) or 5, 10, or 15 mg/kg of Corilagin. The doses of Corilagin used were in reference to the animal experiments of Hau DK’s group [4]. The mice were treated three times per week for four weeks and were then sacrificed.

### Statistical analysis

All data were subjected to statistical analysis and were reported as the mean ± standard deviation. The criterion for statistical significance was taken as P<0.05 using a two-tailed t-test and the count data were tested using chi-square criterion comparing the parameters frequency of parameters. The analyses were performed using SPSS 15.0 software.

## Results

### Corilagin inhibits the growth of ovarian cancer cell lines *in vitro* and *in vivo*

**Figure 2:**
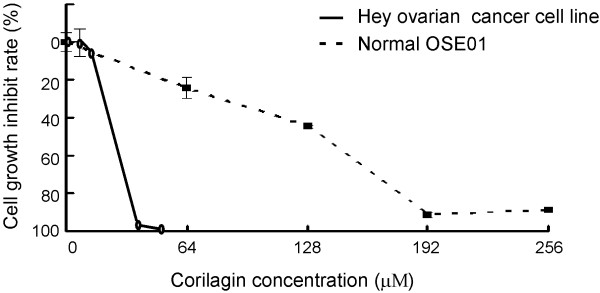
**Corilagin inhibits the proliferation of ovarian cancer cells but not normal OSE cells. **Hey and OSE01 cells were treated with different concentrations of Corilagin for 72 hours. The growth curve was measured by SRB assay repeated three times.

Ovarian cancer cell lines (Hey, SKOv3ip, and HO8910PM) and normal OSE cells (OSE01, OSE02, and OSE03) were used to examine the effects of Corilagin in cell culture. Corilagin demonstrated clear inhibition of ovarian cancer cell growth (the IC50s were 27 μM for SKOv3ip and 28 μM for Hey cells) but had much lower cytotoxicity in normal OSE cells, with IC50s of approximately 160 μM (148 μM for OSE01, 176 μM for OSE02 and 192 μM for OSE03) (Figure 2).

**Figure 3:**
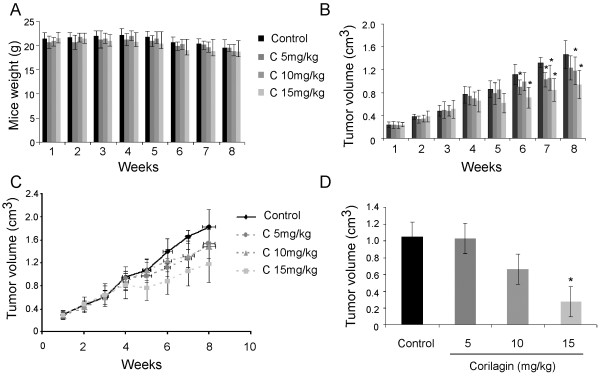
**Corilagin inhibits the growth of SKOv3ip xenograft tumors. **(**A**) Mouse weight measurements during treatment (six to eight mice in each group). (**B**) Xenograft tumor volume measurements during treatment (*P<0.05 compared with control). (**C**) Xenograft tumor growth curve by volume measurement during treatment. (**D**) Final xenograft tumor volumes (*P<0.05 compared with control).

To determine if Corilagin had the same effect *in vivo*, Corilagin was delivered by intraperitoneal injection into mice bearing SKOv3ip xenografts. Mouse weight measurements were not significantly different between the control and Corilagin-treated groups (Figure 3A), but xenograft tumor size was reduced significantly (*P*<0.05) in the Corilagin-treated groups, especially in the 15 mg/kg group, compared with the control group (Figure 3B, C). The final volume measurement of the xenograft tumors (Figure 3D) also showed that the 15 mg/kg Corilagin treatment statistically inhibited tumor growth (*P*<0.05). Thus, the growth of the SKOv3ip xenografts was significantly inhibited by Corilagin treatment.

### Corilagin induces G2 cell cycle arrest and apoptosis

**Figure 4:**
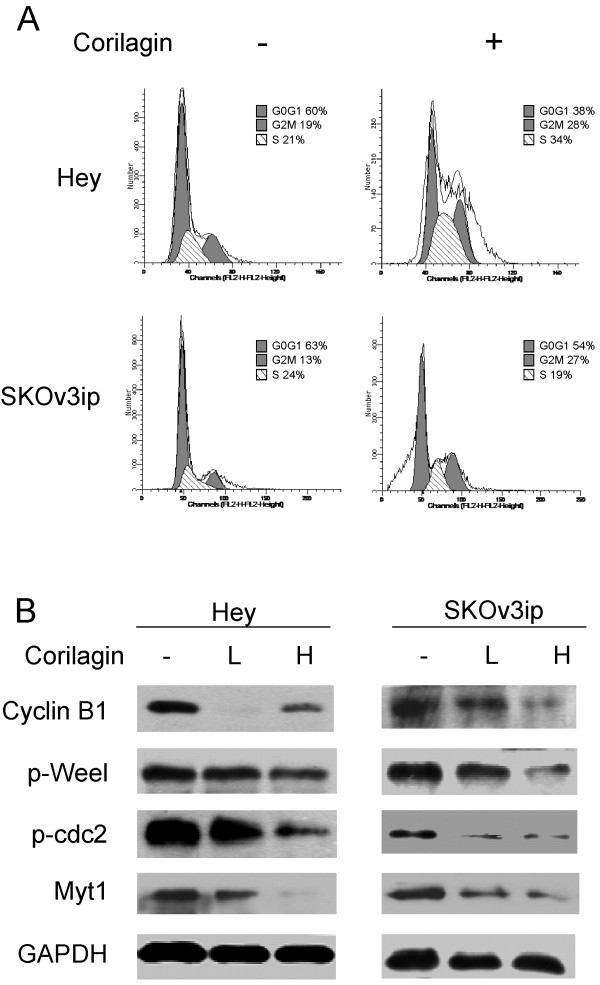
**Corilagin induces G2/M cell cycle arrest and inhibits cell cycle-related proteins. **(**A**) Hey and SKOv3ip cells were treated with Corilagin (40 μM). Cell cycle analyses were performed using flow cytometry after two days of treatment. Cells without treatment were used as the control. The experiments were repeated twice (see Table 1), with the figure depicting a representative experiment. (**B**) Hey and SKOv3ip cells were treated with Corilagin for two days at different concentrations (lower, 20 μM, and higher, 40 μM). Cells without treatment were used as the control. Cyclin B1, p-Weel, p-cdc2 and Myt1 levels were analyzed by Western blotting.

**Figure 5:**
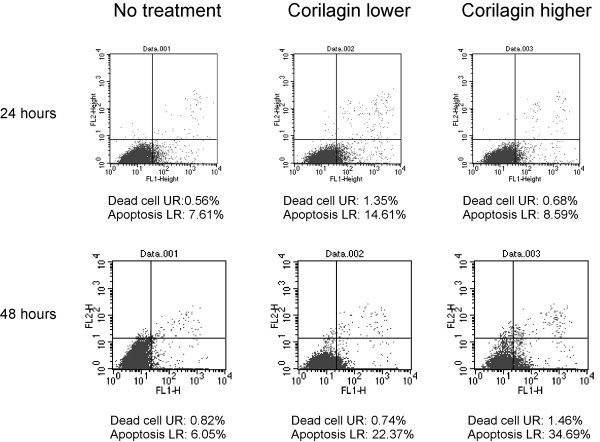
**Corilagin induces cell apoptosis. **Hey cells were treated with Corilagin at different concentrations (lower, 20 μM, and higher, 40 μM); apoptosis was evaluated by flow cytometry at 24 hours (top row) and 48 hours (bottom row) after treatment. Cells without treatment were used as the control. UR: upper-right corner of flow cytometric analysis, LR: lower-right corner of flow cytometric analysis.

When Hey and SKOv3ip cells were treated with Corilagin, the frequency of cells in the G2/M phase was markedly increased compared with the untreated cells (Table 1, Figure 4A). Furthermore, analyses of cell cycle-related proteins suggest that Corilagin arrested ovarian cancer cells in the G2/M phase by down-regulating the expression levels of Cyclin B1, Myt1, Phospho-Weel (p-Weel) and Phospho-cdc2 (p-cdc2) (Figure 4B). Corilagin also induced apoptosis in the ovarian cancer cells. Figure 5 shows that the number of apoptotic Hey cells was significantly increased after 48 h of treatment with Corilagin.

**Table 1:** Corilagin induces G2/M cell cycle arrest in Hey and SKOv3ip cells

		**G0/G1 (%)**		**S (%)**		**G2/M (%)**	
**Cell line**		**Control**	**Corilagin**	**Control**	**Corilagin**	**Control**	**Corilagin**
Hey	1^st ^assay	60	38	21	34	19	28
	2^nd ^assay	55	50	26	29	19	21
SKOv3ip	1^st ^assay	64	47	20	20	16	33
	2^nd ^assay	63	54	24	19	13	27

### Corilagin inhibits the secretion of TGF-β1

**Figure 6:**
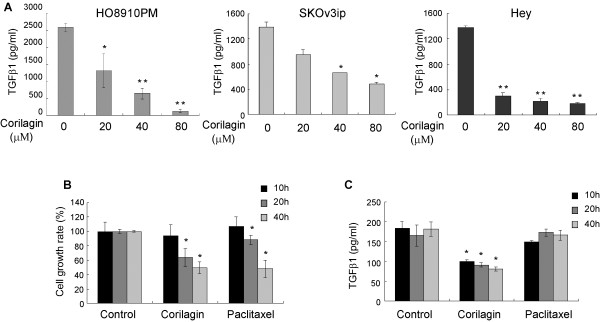
**Corilagin inhibits the secretion of TGF-β1 in ovarian cancer cells. **(**A**) The TGF-β1 secretion of three ovarian cancer cell lines, HO8910PM, SKOv3ip and Hey, was inhibited by Corilagin in a dose-dependent manner. The experiments were repeated three times (*P<0.05 or **P<0.01, compared with the untreated control cells). (**B**) Both Corilagin (40 μM) and Paclitaxel (40 nM) inhibited Hey cell growth. Experiments were repeated three times (*P<0.05 compared with untreated control cells). (**C**) Corilagin, but not Paclitaxel, inhibited the secretion of TGF-β1 in Hey cells. Experiments were repeated three times (*P<0.05 compared with untreated control cells).

Corilagin was reported to inhibit TNF-α secretion [5,6], but TNF-α was unable to be detected by regular ELISA from the culture supernatants of ovarian cancer cells. We tested whether Corilagin could inhibit additional inflammatory factors. Previously, a high concentration of TGF-β was detected in ascites, blood and other bodily fluids of ovarian cancer patients. Using an ELISA, we also found that most ovarian cancer cell lines secrete TGF-β1 into cell culture supernatants, and this secretion increased as the growth rate increased. In this study, we found that TGF-β1 secretion dramatically declined in a dose-dependent manner in the culture supernatants of Hey, SKOv3ip and HO8910PM cells (Figure 6A). Comparing Corilagin with Paclitaxel, a known chemotherapeutic drug for ovarian cancer, Corilagin inhibited both cell growth and the secretion of TGF-β1, while Paclitaxel only inhibited cell growth (Figure 6B, C).

### Corilagin blocks multiple signaling pathways

**Figure 7:**
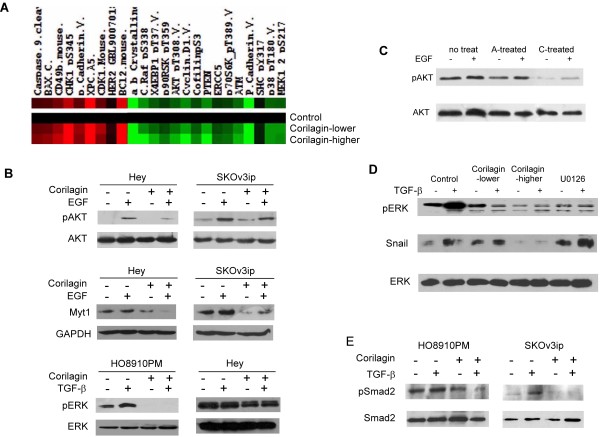
**Corilagin blocks multiple signaling pathways. **(**A**) RPPA analysis of untreated and Corilagin (at lower, 20 μM, and higher, 40 μM, concentrations)-treated HO8910PM cells. Figure presents a small portion of the results. (**B**) Corilagin inhibited pAKT, Myt1 and pERK in Hey, SKOv3ip, or HO8910PM cells. Non-phosphorylated AKT, GAPDH and non-phosphorylated ERK were used as the loading controls. (**C**) Corilagin (as compound C) inhibited AKT signaling in Hey cells, but ethyl brevifolin carboxylate (as compound A) did not. Non-phosphorylated AKT was used as the loading control. (**D**) Corilagin inhibited the expression of pERK and Snail in HO8910PM-Snail cells and the TGF-β-mediated stimulation of pERK and Snail. Non-phosphorylated ERK was used as the loading control. U126, a pERK inhibitor, was used as the positive control. (**E**) Corilagin inhibited TGF-β-mediated pSmad2 expression in HO8910PM and SKOv3ip cells. Non-phosphorylated Smad2 was used as the loading control.

**Figure 8:**
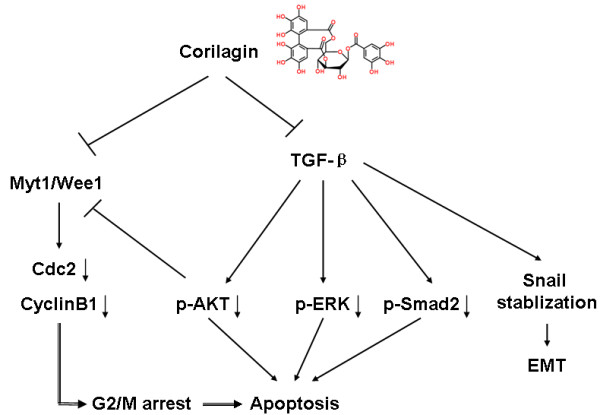
A diagram of the pathways potentially affected by Corilagin.

To understand the anti-tumor mechanisms of Corilagin, we performed a RPPA analysis of untreated and Corilagin (at lower, 20 μM, and higher, 40 μM, concentrations)-treated HO8910PM cells. Figure 7A presents a small portion of the results. The RPPA analysis indicated that several signaling pathways were down-regulated after Corilagin treatment. Western blotting was used to verify these candidates in the HO8910PM, Hey and SKOv3ip cell lines, and we found that Corilagin blocked the activation of multiple signaling cascades, such as pAKT and pERK (Figure 7B, C). Additional candidates from the RPPA analysis will need to be verified. We also observed that Myt1 was down-regulated following treatment with Corilagin either with or without EGF (Figure 7B). We tested two purified extracts from *Phyllanthus niruri* L., ethyl brevifolin carboxylate and Corilagin, but only Corilagin inhibited AKT signaling (Figure 7C). In HO8910PM-Snail cells, Corilagin significantly inhibited pERK and blocked the stimulatory effect of TGF-β on pERK. Corilagin treatment also blocked the upregulation of Snail expression by TGF-β. As an inhibitor of pERK, U0126 could inhibit pERK but had no effect on the expression of Snail (Figure 7D), suggesting that the TGF-β-mediated stimulation of Snail does not occur through pERK. Figure 7E shows that Corilagin blocked pSmad2 with or without TGF-β induction, although SKOv3ip cells were more sensitive than HO8910PM cells to the TGF-β-mediated induction of pSmad2. As a result, Corilagin could be involved in both canonical (Smad) and non-canonical (AKT/ERK) pathways. Figure 8 summarizes the possible signaling pathways that might be affected by Corilagin.

## Discussion

Herbal medicines are currently attracting attention as potential cancer therapeutics and preventive agents. *Phyllanthus niruri* L. is a well-known medicinal plant that has been used as a hepatoprotective, antiviral, antibacterial, analgesic, antispasmodic and antidiabetic medicine; however, there are few reports describing its anti-tumor activity. Our group isolated components of *Phyllanthus niruri* L. by chromatographic fractionation and mass spectrometry. Of the two major isolated components (ethyl brevifolin carboxylate and Corilagin), Corilagin demonstrated better anti-tumor potential and lower toxicity in normal cells [unpublished data, Ming *et al*.]. Corilagin is a gallotannin that has been identified in several plants, including *Phyllanthus niruri* L. [9]. Corilagin has been shown to exhibit versatile medicinal activity including anti-inflammatory effects as well as hepato-protective activity [2,10,11]. Recently, an anti-tumor effect on hepatocellular carcinoma was reported [4]; however, the anti-tumor mechanism is still unclear.

In this study, we confirmed the antitumor effect of Corilagin on ovarian cancer cells and further investigated the mechanism of this effect. Corilagin induced cell cycle arrest at the G2/M stage and enhanced apoptosis in ovarian cancer cells. Cyclin B1, Myt1, Phospho-cdc2 and Phospho-Weel were down-regulated after Corilagin treatment. Importantly, we found that Corilagin inhibited TGF-β secretion into the culture supernatant of all tested ovarian cancer cell lines and blocked the stabilization of Snail induced by TGF-β. The reduction of TGF-β secretion was specific to Corilagin treatment; Corilagin also targeted TGF-β-related signaling molecules, such as pAKT, pERK and pSmads. Other natural products, such as genistein and curcumin, can also alter the TGF-β pathway. Both of these agents can abrogate the enhancement of u-PA levels induced by TGF-β1 and also inhibit the TGF-β1-induced synthesis of fibronectin [12], inferring that some natural products have the potential to be effective in the treatment of cancer.

G2/M checkpoint-based anti-cancer strategies have focused on targeting and inactivating the G2/M checkpoint, thus forcing the cancer cells into mitosis with increased DNA damage and finally into mitotic catastrophe and cell death. The Cyclin B/cdc2 complex performs an important function in controlling the G2/M phase by rapidly phosphorylating the target protein to induce progression into the M phase [13,14]. The phosphorylation and dephosphorylation of specific amino acids in cdc2 are responsible for the control of G2/M cell cycle progression by the Cyclin B1/cdc2 complex [13,14]. More specifically, in the G2 phase, cdc2 is phosphorylated at Thr14 and Tyr15 by the protein kinases Myt1 and Wee1, thereby converting it into an inactive precursor. Consistent with these reports, in the present study, we observed that Corilagin decreases the protein level of Cyclin B1, p-cdc2 (Tyr15) in both Hey and SKOv3ip cells, which might be the molecular mechanism responsible for Corilagin’s efficacy in inducing G2/M arrest. We also observed down-regulation of p-Wee1 (Ser642) and Myt1 in Hey and SKOv3ip cells, indicating that the efficacy of Corilagin in inducing G2/M arrest in ovarian cancer cells is possibly due to the down-regulation of cdc2 and Cyclin B1 through Wee1 and Myt1 regulation.

Akt is suggested to function as a G2/M initiator. The activity of PI3K/Akt is required at multiple points during the cell cycle. Downstream functions of the PI3K/Akt pathway during G2/M transitions may include inhibition of the Chk1 G2 checkpoint protein or activation of cdc25C, which promotes cdc2 activation and entry into mitosis in primary oocytes from the starfish *Asterina pectinifera*[15]. Akt was reported to inhibit Myt1 through Akt-dependent phosphorylation and down-regulation at the G2/M transition [16]. In the present study, we observed that Corilagin inhibited both pAKT (Ser473) and Myt1 expression in Hey and SKOv3ip cells after stimulation with EGF, suggesting that the inhibition of Akt/Myt1 also contributes to the G2/M arrest resulting from Corilagin treatment. Further studies will be required to support these assumptions and to determine the role of upstream events, such as Chk1 and Chk2, in ovarian cancer cell responses to Corilagin.

Corilagin has been reported as a TNF-α-releasing inhibitor in inflammatory scenarios [5,6]. In this study, we observed that the secretion of TGF-β was inhibited by Corilagin in a dose-dependent manner in all ovarian cancer cells evaluated, indicating that Corilagin also disturbed the expression and efficacy of TGF-β. Our results further demonstrated that Corilagin not only targets the classical Smad pathway *via* pSmad2 but also down-regulates MAPK signaling. The thing that most intrigued us is that Corilagin treatment induced a dramatic decline in the expression of the Snail protein, especially at higher doses, which indicates that Corilagin not only exerts its effects on cell cycle control but also contributes to epithelial-mesenchymal transition (EMT) in ovarian cancer.

As with all cancer cells, ovarian cancer cells undergo an EMT to disseminate within the intraperitoneal cavity or metastasize to distant sites [17]. TGF-β signaling plays a critical role in ovarian cancer EMT and metastasis. Ovarian cancer is thought to arise from normal ovarian surface epithelium (OSE). TGF-β has been shown to inhibit human OSE proliferation and induce apoptosis, which may prevent the over-proliferation of cells during a normal ovulatory cycle [18]. Although TGF-β can act as a tumor suppressor by inhibiting cell proliferation in the early stages of tumor development, it can also promote metastasis in various cancer models [19,20]. It appears that at later stages, cancer cells protect themselves and tend to acquire increasing resistance to TGF-β growth inhibitory signals, which is an important reason for the shift of TGF-β from tumor suppressor to tumor promoter [21]. Much remains to be elucidated about how TGF-β contributes to ovarian cancer progression, particularly in the regulation of EMT. A high concentration of TGF-β has been detected in ascites, blood and other bodily fluids of ovarian cancer patients [22]. When ovarian cancer cells were cultured, various TGF-βs, including TGF-β1, TGF-β2 and TGF-β3, induced pro-matrix metalloproteinase (MMP) secretion, the loss of cell-cell junctions, down-regulation of E-cadherin, up-regulation of N-cadherin, and the acquisition of a fibroblastoid phenotype, all of which are consistent with EMT [23-25]. In addition, our recent studies identified that TGF-β is the most important inflammatory factor in ovarian cancer. TGF-β stabilizes the protein level of Snail, an inducer of EMT, and further enhances Snail expression when combined with other inflammatory factors (Jin *et al.* unpublished data). However, how Corilagin has this effect on TGF-β and thus undermines the stability of Snail still needs to be elucidated.

TGF-β binds to type I (ThRI) and type II (ThRII) receptors. Upon ligand binding to ThRII, ThRI is activated and phosphorylates the receptor-regulated Smads. The phosphorylated receptor-regulated Smads then bind to the co-Smad, Smad4, and translocate to the nucleus to modulate gene expression. TGF-β also initiates Smad-independent pathways, including those mediated by the mitogen-activated protein kinase family members (TAK1, extracellular signal-regulated kinase, p38, and c-Jun-NH2-kinase) and phosphatidylinositol 3-kinase [26]. In this study, we found that Corilagin not only inhibits the secretion of TGF-β but also blocks the TGF-β-related signaling proteins pSmads, pAKT, and pERK (Figure 8). Our research provides evidence that TGF-β/Smad/AKT/ERK signaling is the target of Corilagin and that this herbal medicine could be an effective ovarian cancer therapeutic agent.

## Conclusions

Corilagin is a major active component with anti-tumor activity from *P. niruri* L. Our results indicated that Corilagin distinctly inhibited the growth of ovarian cancer cells *in vitro* and *in vivo*, while displaying low toxicity against normal cells. More interestingly, Corilagin inhibited TGF-β secretion and blocked the stabilization of Snail that is induced by TGF-β; Corilagin blocked the activation of both canonical Smad and non-canonical ERK/AKT pathways. Corilagin, therefore, acts as a natural, effective therapeutic agent against the growth of ovarian cancer cells via targeted action on the TGF-β/AKT/ERK/Smad signaling pathways.

## Abbreviations

*P. niruri* L: *Phyllanthus niruri* L; OSE: Ovarian surface epithelial; SRB: Sulforhodamine B; RPPA: Reverse phase protein array; EMT: Epithelial-mesenchymal transition; p-Weel: Phospho-weel; p-cdc2: Phospho-cdc; MPLC: Medium Pressure Liquid Chromatography; HPLC: High Performance Liquid Chromatography.

## Competing interests

These authors report no financial or intellectual conflicts of interest regarding this study.

## Authors’ contributions

LJ, HJ and JZ conducted the cell culture, signaling analysis and animal experiments. LJ and HJ prepared the manuscript. LJ and HJ performed the statistical analysis. YL assisted with the RPPA analysis. LC and YM purified and provided the Corilagin. YY and YM conceived the study and gave final approval of the manuscript. All authors read and approved the final manuscript.

## Pre-publication history

The pre-publication history for this paper can be accessed here:

http://www.biomedcentral.com/1472-6882/13/33/prepub
